# Changes in Phylogenetic and Functional Diversity of Ciliates along the Course of a Mediterranean Karstic River

**DOI:** 10.3390/microorganisms10122493

**Published:** 2022-12-16

**Authors:** Vesna Gulin Beljak, Antonija Kulaš, Guillaume Lentendu, Barbara Vlaičević, Marija Gligora Udovič, Mirela Sertić Perić, Fran Rebrina, Petar Žutinić, Sandi Orlić, Renata Matoničkin Kepčija

**Affiliations:** 1 Department of Biology, Faculty of Science, University of Zagreb, Rooseveltov Trg 6, 10000 Zagreb, Croatia; 2 Laboratory of Soil Biodiversity, Institute of Biology, University of Neuchatel, Rue Emile Argand 11, CH-2000 Neuchatel, Switzerland; 3 Department of Biology, Josip Juraj Strossmayer University of Osijek, Ulica cara Hadrijana 8A, 31000 Osijek, Croatia; 4 Ruđer Bošković Institute, Bijenička Cesta 54, 10000 Zagreb, Croatia

**Keywords:** eukaryotes, karst, environmental filtering, freshwater, phagotrophic, competitive exclusion

## Abstract

Ciliates are a group of phagotrophic protists found in a wide variety of ecosystems. This study builds on recent studies of ciliates in the Krka river and investigates changes in the phylogenetic and functional diversity of ciliates in biofilm to predict the phylogenetic and functional structure of ciliates in other karstic rivers. Biofilm samples were collected from four representative locations: upstream (Krka spring), midstream (Marasovine), and downstream (Roški slap, Skradinski buk) of the Krka river to test for differences in phylogenetic and functional diversity of ciliates in relation to location and positioning on tufa stones (light/dark-exposed side of tufa stone). Our results showed that Krka spring had higher phylogenetic species variability, lower phylogenetic diversity, and lower functional richness than Skradinski buk, suggesting phylogenetic overdispersal at Krka spring. This could be due to environmental filtering, competitive exclusion, or a combination of these factors. As the first study of its kind in the Mediterranean, our results shed light on the phylogenetic and functional diversity of ciliates in karst ecosystems and provide a basis for future ecological and conservation efforts.

## 1. Introduction

Ciliates (Ciliophora) are a widely distributed group of phagotrophic protists [1]. Because of the various food they consume and the different feeding mechanisms, ciliates are considered essential elements in the trophic webs of freshwater ecosystems [2]. Their ubiquity, abundance, and sensitivity make them excellent bioindicators in a number of different environments, including karst environments [3,4,5]. Not only are they widely distributed in various types of communities (benthos, biofilm, and plankton) in tufa-depositing ecosystems, but they also show a rapid response in terms of their taxonomic and functional diversity to changing conditions, such as revitalization of tufa barriers [6,7].

Environmental filtering and competitive exclusion have been recognized as main drivers of phylogenetic and functional diversity of protists in different environments [8,9,10]. While competitive exclusion is thought to be stronger at the local scale (alpha diversity), environmental filtering is considered more likely to influence regional community patterns (beta diversity) [11,12,13]. These two processes assumingly have opposing effects on community structure with interspecific competition suppressing niche overlap in co-occurring species on the one hand, and environmental filtering promoting ecological similarity on the other [14,15]. When ecological niches are phylogenetically conserved, these two processes are expected to lead to a distinct phylogenetic community structure: competition results in phylogenetic overdispersion (i.e., divergence), and environmental filtering results in phylogenetic clustering [16]. These effects are also reflected in functional aspects, with convergence or divergence in functional diversity [16].

Incorporating multiple diversity indices (beyond taxonomic diversity) through an integrative approach is now considered essential for a comprehensive understanding of ecosystems and their conservation [17]. While phylogenetic diversity can shed light on how evolutionary history has shaped present-day microbial communities, functional diversity allows linking environmental responses and ecosystem functions [18]. Phylogenetic diversity (usually expressed by Faith’s PD—the sum of branch lengths of a phylogenetic tree [19]) is a critical component of modern ecology and is considered one of the best predictors of ecosystem function in different ecosystems [20]. Greater PD implies greater variation in functional traits because species in an assemblage are expected to encompass greater spans of independent evolutionary time, allowing them to accumulate greater variation in ecological traits and provide complementary functions [21]. In addition to PD, PSV (phylogenetic species variability index [22]) is one of the most commonly used metrics to illustrate phylogenetic diversity. It quantifies how phylogenetic relatedness reduces the variance of a hypothetical neutral trait shared by all species in a community, indicating phylogenetic dispersion [22].

In addition to phylogenetic alpha diversity, phylogenetic beta diversity measures such as UniFrac [23,24] also provide a valuable source of information as they use similarities and differences between species [25,26]. This additional information makes phylogenetic beta diversity measures more effective in representing ecological patterns, determining whether microbial protist communities (ciliates) are significantly different, identifying individual lineages that contribute to these differences, and revealing broad-scale patterns that span many environmental samples [24,27,28].

Functional diversity (FD) is generally based on species’ functional traits, i.e., traits of organisms that influence not only their fitness but also their response to the environment and/or their impact on ecosystem properties community [29]. Higher functional diversity is related to improved ecosystem resilience, which is the ability of a system to absorb shocks, reorganize, and maintain the same structure and function community [30]. One of the key components of functional diversity is functional richness (FRic), defined as the amount of niche space occupied by species within the community [31]. The results of Mouchet et al. [32] show that FRic is highly informative in detecting both environmental filtering and competitive exclusion, and that it more accurately represents the change in dimensionality of functional space caused by community structure than other metrics of functional diversity because it is based on presence/absence rather than abundance data.

According to Weisse [33], the functional categorisation of aquatic ciliates needs to be as detailed as possible, as the use of broad categories and collective terms is not very useful for functional diversity-related analyses. For example, grouping ciliates into (too) broad categories of food source (algivorous, bacterivorous, omnivorous) may be one of the reasons why Van Wichelen et al. [34] did not detect differences in functional composition and diversity between 66 turbid and clear-water shallow lakes in north-western Europe. Therefore, functional traits in this study were assigned in as much detail as possible according to Foissner’s classification [3,35] and the latest revision by Adl et al. [36]. Ciliates were assigned to categories for the following functional traits: motility (euplanktonic; motile; semi-sessile; sessile; unknown), life form (colonial; solitary; unknown), mode of locomotion (crawling/creeping; drifting; free-swimming; gliding; jumping; rotating; unknown), habitat preference (active sludge; active sludge, soil; anaerobic mud; brackish; lentic and lotic (freshwater); marine; soil; symbiotic), food source (algae; algae, bacteria; algae, cyanobacteria; algae, diatoms; astomata; bacteria; cyanobacteria; diatoms; fungi; histophagous; omnivorous; parasitic; phagotrophic protists, small metazoans; unknown), and feeding strategy (filtration; filtration, facultative predation; predation; unknown).

This set of traits has proven useful in several recent studies concerning ciliates [37,38] and in assessing the functional response of ciliates to stream revitalization at the Skradinski buk tufa barrier, Krka river [7]. Assignment of functional traits, i.e., categorisation, was based on coding of data obtained after careful review of the literature. For example, we found literature data relevant to habitat preference for 11 species of the genus *Vorticella*, and more than 50% of these species occurred in active sludge in addition to lotic and lentic habitats, so the genus *Vorticella* was assigned “active sludge”. This simply means that the genus occurs in active sludge in addition to the predominant freshwater habitats. The reason for distinguishing “active sludge” as a separate trait was simply to highlight that some of the genera detected in the study also occur in nutrient-rich habitats and can reach high abundances (in contrast to usually oligotrophic tufa-depositing systems). This reasoning was also applied for the “soil” and “marine”. The same coding principle was applied to all other categories (food source, feeding strategy, motility, mode of locomotion, and life form). If we found that more than 50% of the species within the genus fed on something other than just two types of food, they were classified as “omnivorous”; otherwise, their food source was differentiated into two dominant sources, e.g., “algae, bacteria”.

This study builds on the results of Kulaš et al. [5] who showed that the taxonomic diversity of ciliates studied using a combined approach of traditional microscopic analysis and environmental DNA (eDNA) metabarcoding can be a good bioindicator of karstic environments such as the Krka river. It aims to investigate the changes in phylogenetic and functional diversity of ciliates in the Krka river biofilm to predict phylogenetic and functional structure of ciliates in other karstic environments. Since previous results showed significant differences in ciliate community structure between upstream and downstream sampling locations, our goal was to test whether the location effect was also reflected in phylogenetic and functional diversity. We hypothesised that both phylogenetic and functional diversity of ciliates would increase at downstream, leading to significant differences with regard to location but also in relation to positioning on tufa stone (light/dark-exposed side of tufa stone), as microhabitat complexity has already been recognised as one of the main factors driving taxonomic diversity of protists in tufa-depositing environments, including the Krka river [6,7]. In addition to the selected metrics, our study also provides insights into the composition of the ciliate community in terms of functional groups, thus providing a comprehensive and valuable database of assigned functional traits. Being the first to examine the phylogenetic and functional diversity of ciliates in a Mediterranean karstic river, our study lays the foundation for future research on this topic and opens a new opportunity to more frequently include ciliates in monitoring and conservation plans for various karst freshwater habitats, not only for tufa barriers.

## 2. Materials and Methods

### 2.1. Study Area

**Figure 1:**
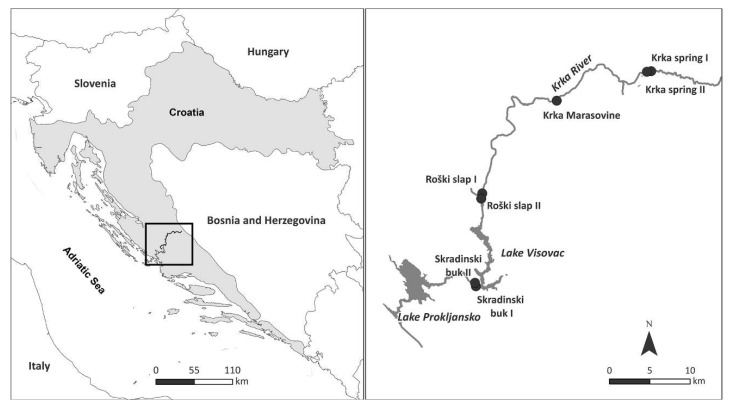
Map of the four sampling locations (Krka spring, Marasovine, Roški slap and Skradinski buk) situated at the Krka river, Croatia (Krka spring, Roški slap and Skradinski buk were sampled on two representative habitats—marked with “I” and “II”).

The Krka river is a 73 km long river in the Dinaric region of Dalmatia, Croatia. It is a specific karstic river with a strong connection of surface and groundwater, characterised by tufa barriers formed through deposition of CaCO_3_ under specific physical and chemical conditions [39,40]. The headwaters of the Krka river are located near the Dinara mountain and consist of three permanents springs that flow through the Knin Karst Polje after the headwaters [41,42]. Along the composite valley of the Krka river, there are seven major tufa barriers forming waterfalls, in the downstream direction [43]. Some of them form lacustrine sections in the river, influencing the dynamics of the river by creating segments with alternating lotic and lentic parts. The length of the Krka river from the source to the last tufa barrier Skradinski buk is 49 km. Thereafter, the river flows into the Adriatic Sea near the town of Šibenik in a brackish estuary about 25 km long [43]. The four sampling locations (Krka spring, Marasovine, Roški slap, Skradinski buk) were chosen to represent the upstream (Figure 1), midstream, and downstream sections of the river. Sampling locations were adapted from [5], while Krka spring (I and II), Roški slap (I and II) and Skradinski buk (I and II) were sampled on two representative habitats.

### 2.2. Sampling Procedure

Sampling was conducted from 21 to 23 September 2017 along the course of the Krka river. At each sampling location, three individual sub-locations were chosen (at least 10 m apart), with each successive sub-location selected upstream of the previously sampled one. Transverse samples were exceptionally taken at those locations where longitudinal samples were not possible due to waterfalls. Pooled samples were collected at each sub-location, consisting of at least five randomly selected tufa stones which were scrubbed and/or brushed with a new toothbrush to collect biofilm from the light-exposed and dark-exposed sides of the tufa stones and rinsed with water. Samples of the biofilm were stored for molecular analysis in Falcon tubes (50 mL) without added preservatives, kept on ice during transport to the laboratory, and stored at −20 °C until further processing. For physicochemical analysis, in situ measurements of water temperature, pH, conductivity (EC), oxygen concentration and saturation were performed using a portable multimeter (Hach HQ40d, Germany). Samples for water chemistry analysis were collected simultaneously with the biological samples and stored at −20 °C until processing in the laboratory. The following parameters were quantified according to compliance monitoring standards (CEN-EN 15708, 2009): nitrites (NO_2_^−^-N), nitrates (NO_3_^−^-N), ammonium (NH_4_^+^-N), phosphates (PO_4_^3−^-P), total nitrogen (TN), silicon dioxide (SiO_2_), total inorganic carbon (TIC), dissolved inorganic carbon (DIC), total organic carbon (TOC), and dissolved organic carbon (DOC).

### 2.3. DNA Extraction and PCR Reaction

DNA extraction, PCR reaction, and bioinformatic processing were performed as previously described in [5]. DNA was isolated from the frozen epilithic biofilm. The first step was to remove excess water by centrifugation (4000× *g* for 1 min). The DNeasy PowerSoil Kit (Qiagen, Germany) was used for DNA isolation, following the manufacturer’s instructions. In the final step of the isolation, a minor modification was made by adding 60 µL of sterile, DNA-free, PCR-grade water instead of the C6 solution from Qiagen. The yield and quality of extracted DNA was measured using a spectrophotometer (BioSpec Nano, Shimadzu, Japan). Environmental DNA was amplified using the universal hypervariable V9 region of the SSU rRNA gene (approximately 130 base pairs) with primer pairs 1391F (5′-GTACACACCGCCCGTC-3′) and EukB (5′-TGATCCTTCTGCAGGTTCACCTAC-3′) designed by [44]. PCR products were assessed by visualisation on a 1% agarose gel according to the protocol of [45,46]. Sequencing libraries were prepared using the NEB Next^®^ Ultra™ DNA Library Prep Kit for Illumina (NEB, Ipswich, MA, USA). Libraries were sequenced on an Illumina NextSeq platform generating 150-bp paired-end reads (SeqIT GmbH & Co. KG, Kaiserslautern, Germany).

### 2.4. Bioinformatics and Statistics

Raw Illumina reads were demultiplexed using Cutadapt v1.18 [47], removing barcodes in the 5´ to 3´ combination, and then processed using the DeltaMP pipeline v0.3 (https://github.com/lentendu/DeltaMP, accessed on 30 October 2018.). In the final steps, sequences were grouped into OTU using SWARM v2 [48] and the global pairwise alignments of VSEARCH’s were used for taxonomic assignment with the Protist Ribosomal Reference (PR2) database v.4.12.0 and a threshold value of 80% identity [49]. A consensus taxonomy with a 60% threshold was created for Operational Taxonomic Units (OTUs) with multiple best matches to different taxonomy in the database. To keep only protist OTUs, OTUs assigned to the following taxa were removed: Streptophyta, Metazoa, Fungi, unclassified Archaeplastida, unclassified Eukaryota, and unclassified Opisthokonta. Ciliophora was one of the dominant groups of protists in the dataset [5]. Using Vsearch (v. 2.17.0), only the sequences of Ciliophora were extracted for all subsequent statistical and phylogenetic analyses. OTUs of ciliates were also removed if they were not phylogenetically placed within the known eukaryotic clades using the evolutionary placement algorithm [50] reduced to the V9 region. Phylogenetic trees were inferred using multiple sequence alignments of OTU representatives generated with MAFFT v7.490 [51], and picking the highest likelihood tree inferred with IQtree v1.6.12 [52] using the GTR+FO+G model.

Statistical analyses were conducted in R v.4.1.2 [53]. To avoid bias in alpha and beta phylogenetic diversity indices due to unequal sequencing depth of the main taxa and subtaxa in the different samples, read counts were normalized using the center-log ratio transformation [54]. Phylogenetic species variability index—PSV [22] and PD- phylogenetic diversity [55]—were estimated with “picante” package v.1.8-2 [56]. PSV is statistically independent of species richness and reaches a value of 1 when all species are unrelated and zero as species become more related.

Beta diversity was estimated using the unweighted (based on presence/absence) and weighted (based on relative abundance) UniFrac distance [23] with “phyloseq” package v.1.36-0 [57]. To test significance and to detect individual and combined effects of locations and exposed sides, beta diversity was constrained by the PERMANOVA permutation test. Non-metric multidimensional scaling (NMDS) was used to visualise beta diversity with significant environmental variables fitted to the ordination using the *envfit* function of the “vegan” package v2.5-7.0 [58]. The fit (R^2^) of each variable to the ordination was assessed with a Monte Carlo analysis of 10,000 permutations.

Functional diversity of ciliates was quantified using functional richness metric (FRic), which calculates the volume of the multidimensional convex hull encompassing all ordinated trait values in a set [59]. This metric captures the total amount of variation in trait values, making it conceptually analogous to Faith’s PD [60]. FRic was calculated using the “FD” package v.1.0-12. [61,62]. A detailed overview of the criteria for classification can be found in Supplementary Table S1.

Proportions of ciliate functional groups at each sampling location were visualised using packages “ggrepel” v.0.9.1. [63] and “tidyverse” v.1.3.1. [64].

Tukey’s HSD parametric test was applied to test location and side effect (for each location pair and side pair) for phylogenetic alpha diversity indices (PD and PSV) and functional richness metric (FRic), separately. The results were visualised as different letters above boxplots using “ggplot2” v.3.3.5 [65].

The demultiplexed raw reads were deposited in the Sequence Read Archive of ENA and are publicly available under project number PRJEB39359.

## 3. Results

### 3.1. Sequencing

A total of 32,361,941 sequencing reads were yielded from the remaining 39 samples into 394,492 OTUs [5]. After extraction of low abundance reads, a total of 31,907,533 high-quality reads were clustered into 82,918 OTUs. In the end, a total of 5,413,607 protist reads were found in 11,295 OTUs. Within the protist OTUs, only 466,344 reads were taxonomically assigned to Ciliophora with 3724 OTUs. The Ciliophora reads were extracted from the dataset and further analysed in detail. The number of families, genera, and species of ciliates identified were detailed in an earlier study [5].

### 3.2. Ciliate Phylogenetic and Functional Diversity Metrics

**Figure 2:**
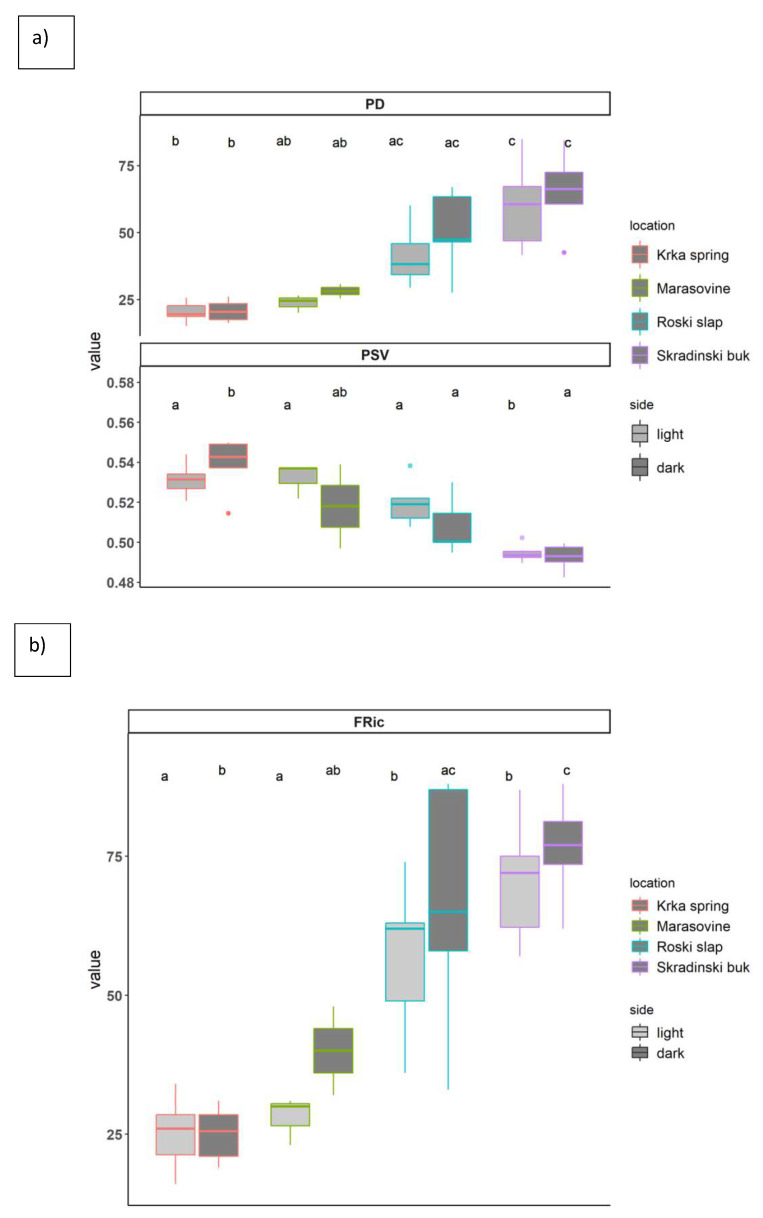
Changes in phylogenetic alpha diversity indices for PD—phylogenetic diversity and phylogenetic species variability index—PSV (**a**) and functional richness—FRic (**b**) visualised by boxplots for each sampling side and location; the horizontal thick black band represents the median values, and the boxplot margins indicate first and third quartiles, different letters above boxplots indicate significant differences among sampling side and location based on Tukey’s HSD test. The lighter grey colour denotes light-exposed samples, whilst darker grey colour denotes dark-exposed samples.

The results of Tukey’s HSD parametric test confirmed significant differences in PD, PSV and FRic with regard to the location (Figure 2a,b), but not with regard to side effect (light/dark exposure). At the upstream location of Krka spring had significantly lower values of PD and FRic in comparison to the downstream-most location Skradinski buk while the opposite was true for PSV values. The detailed results of Tukey’s HSD parametric test can be found in Supplementary Table S2.

**Figure 3:**
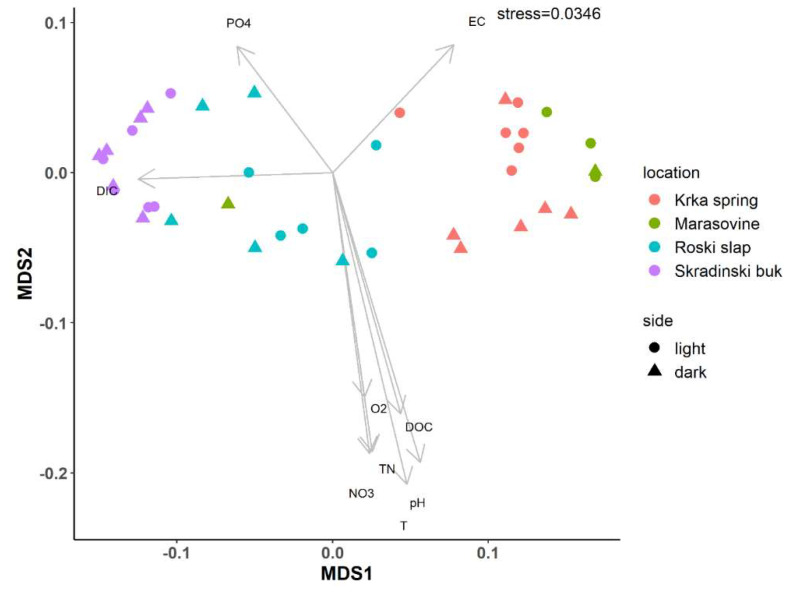
Non-metric multidimensional distance scaling (NMDS) analysis of the Unifrac distance indices of the ciliate community at OTU level with environmental parameters (DIC = dissolved inorganic carbon, DOC = dissolved organic carbon, EC = conductivity, N-NO_3_, O_2_ = oxygen saturation, pH, PO_4_ = phosphates, T = temperature, TN = total nitrogen).

The community composition at sampling side and location was analysed via NMDS of the Unifrac distance measure for ciliates (Figure 3). There was a separation of sampling location, specifically a separation of the upstream and downstream parts of the river section. This was also confirmed by the PERMANOVA test, where the location effect showed significance (*p* = 0.003), while side (*p* = 0.987) and combined (*p* = 0.403) effects were not significant. NMDS ordination showed that environmental parameters consistently influenced the beta diversity of the ciliate community, resulting in a clear separation of upstream and downstream samples along the NMDS1 axis. Environmental parameters at the upstream locations were characterised by higher phosphate values (Krka spring) and higher electrical conductivity (Marasovine). Roški slap samples were separated between the upstream and downstream river section. Samples of Roški slap associated with the upstream samples were characterised by higher phosphate values, while the downstream samples were characterised by higher nitrate values, DOC and DIC. Higher values of DIC also characterised the samples at Skradinski buk, the downstream part of the river (Supplementary Table S3 [5]). The main parameters that showed a significant (*p* < 0.01) positive correlation with the MDS1 axis were pH, temperature, oxygen saturation, nitrates, TN, and DOC, while DIC (*p* < 0.05) showed a significant negative correlation with both axes MDS1 and MDS2 (Supplementary Table S4).

### 3.3. Community Composition with Regard to Ciliate Functional Groups

**Figure 4:**
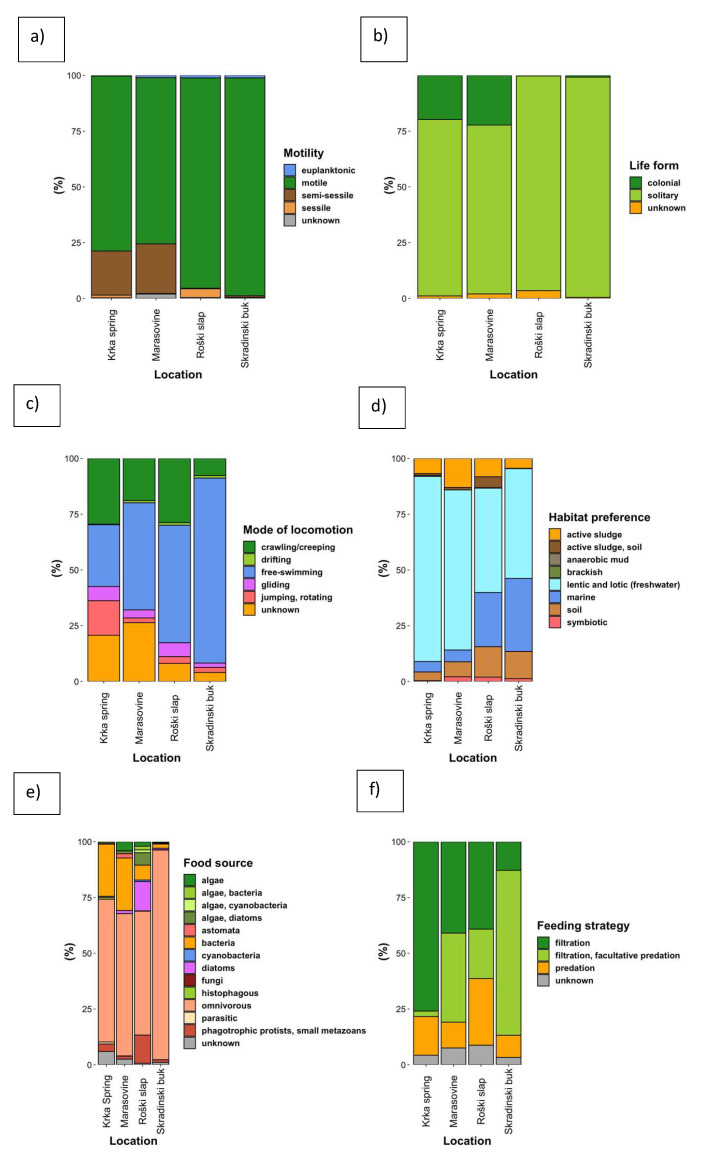
Proportion of functional groups with regard to (**a**) motility; (**b**) life form; (**c**) mode of locomotion; (**d**) habitat preference; (**e**) food source; (**f**) feeding strategy at each of the four sampling locations: Krka spring, Marasovine, Roški slap and Skradinski buk.

There were differences in the proportion of certain functional groups of ciliates upstream and downstream of the Krka river (Figure 4). In terms of motility and mode of locomotion, the upstream locations, i.e., Krka spring and Marasovine, had a higher proportion of semi-sessile and sessile species than the downstream location (Skradinski buk) (Figure 4a). Most of these sessile ciliates were colonial species, abundant only at the upstream locations (Figure 4b). As for the motile ciliates, most categories, in terms of mode of locomotion, were more or less evenly represented at the upstream locations, while the free-swimming ciliates dominated at Skradinski buk, where they reached a higher proportion than at the other sampling locations (Figure 4c). Skradinski buk was also the location with the highest proportion of euplanktonic ciliates.

In terms of habitat preference, the upstream locations were again more diverse than the downstream ones; however, Skradinski buk was the only location with brackish taxa and the one with the highest count of OTUs belonging to marine taxa (Figure 4d).

In terms of food source and feeding strategy, the same pattern emerged with a more diverse community at upstream locations. Skradinski buk had the highest proportion of omnivorous taxa in the community. These taxa also characterised the upstream locations; however, a substantial proportion of bacterivorous ciliates and those feeding on algae and diatoms was also found (Figure 4e). There were also differences in community composition in terms of feeding strategy. Krka spring was dominated by filter-feeders, while the community at Skradinski buk was dominated by taxa that were both filter-feeders and facultative predators (Figure 4f).

## 4. Discussion

The main finding of our study is that the upstream location, i.e., Krka spring, had the most divergent community and consisted of distantly related taxa. Under the assumption of phylogenetically conserved ecological niches, it could be hypothesised that this phylogenetic overdispersion resulted from competitive exclusion rather than environmental filtering. However, this hypothesis is plausible if environmental filtering and competitive exclusion have mutually exclusive effects on the community. Recent studies have instead suggested that environmental filtering and competitive exclusion are not strictly opposing processes but may act in parallel or even interact [10,16]. Therefore, the complex relationship between these two processes should be considered.

We hypothesise that the environmental conditions at Krka spring, e.g., lower nutrient concentrations (except for phosphates) and lower discharge rates, favoured better competitors, such as semi-sessile and sessile taxa, that were predominantly colonial, bacterivorous filter feeders. Some studies [66,67] have already shown that sessile and semi-sessile ciliates (as many heterotrichs) were much better competitors than free-swimming ciliates because of their lower weight-specific respiratory rates, which allow them to better reduce their metabolism upon starvation. Most of these sessile ciliates belonged to peritrichs, which have higher growth rates and are less likely to be washed away [33,68]. Peritrichs are also very successful filter feeders and feed primarily on bacteria. Kusuoka and Watanabe [68] reported increased competition within the community when peritrich abundance increases, as large colonies generate greater currents and can potentially filter more bacteria. Significantly lower discharge values at Krka spring than at the downstream locations Roški slap and Skradinski buk (detailed discharge values can be found in [5]) confirm this. The lower discharge appears to be favourable for sessile and semi-sessile ciliates, which tend to be more firmly anchored in the substrate and therefore more unlikely to detach. Semi-sessile filter feeders proliferate in patches that provide shelter but also allow circulation of water and food [69].

It is already known that phosphate concentration influences the biofilm functional trait composition [70,71,72]. Of all sampled locations, Krka spring had the highest phosphate concentration (Supplementary Table S3), which likely originated from groundwater. Karst springs in the Krka river basin are often associated with inflow of groundwater originating from the mountain Dinara [73]. Groundwaters in the Croatian and Slovenian karst areas rarely contain more than 0.1 mg L−1 of phosphates unless they are polluted [74]. Although the various forms of dissolved phosphorus are mostly derived from natural N and P cycles from river-born material, they can also be a result of anthropogenic activities [75,76]. Phosphates, along with nitrates, are the predominant forms in total N and P fluxes. It is likely that the high phosphate concentration at Krka spring originated from groundwater and promoted algal and microbial growth supporting colonial, bacterivorous, filter-feeding ciliates. Baković et al. [77] already found that karst underground habitats of Croatia and Bosnia and Herzegovina are a hotspot of protist biodiversity; therefore, it is likely that the influx of groundwater served as a mechanism of ciliate colonization at Krka spring. Interestingly, Kulaš et al [78] recorded diatom species (Aulacoseira granulata (Ehrenberg) Simonsen and Navicula cryptotenella Lange-Bertalot) at Krka spring that tolerate higher phosphate concentrations. This supports the idea that phosphate concentration, along with discharge rate, may be one of the main factors determining the ciliate functional community composition at Krka spring.

Although our results showed that the positioning on tufa stone (light/dark-exposed side of tufa stone) was not significant for the tested phylogenetic and functional diversity metrics, microhabitat conditions and complexity have still been recognized as one of the most important drivers of taxonomic diversity of protists (including ciliates) in tufa-depositing environments and in the Krka river basin itself [5,6,7]. Lentendu and Dunthorn [10] showed that local heterogeneity can influence competitive exclusion, and that competitive exclusion has a greater effect in less heterogenous habitats. Snyder and Chesson [79] also found that high local heterogeneity promotes coexistence of competing species. Therefore, it is possible that Krka spring consisted of more homogeneous microhabitats and favoured more homogeneous environmental conditions than downstream locations. In fact, NMDS results showed that downstream locations were characterised by higher nitrate, DOC, and DIC values, suggesting that these locations may be considered nutrient-rich microenvironments that support the development of heterogeneous microhabitats where the strength of mutual exclusion is much lower [10]. Although the positioning of tufa stone may not have been the correct element to measure local heterogeneity because it did not show a significant effect, local heterogeneity may still be a factor influencing ciliate phylogenetic and functional diversity. Results of Gulin et al. [6,7] showed that the taxonomic and functional diversity of ciliates is highly dependent on methodology and varies significantly between natural and artificial substrates.

Local heterogeneity was also reflected in functional richness values. Higher FRic in the downstream locations of the Krka river are likely related to local microhabitat complexity, as the abundance of various tufa-depositing forms is much higher at the downstream locations, especially Skradinski buk [80]. Skradinski buk is the final tufa barrier located about 13 kilometres downstream from Roški slap and about 50 kilometres downstream from the source. Further downstream, the Krka river is under maritime influence, with brackish elements [80]. This is also evident from our results, which show that Skradinski buk was the only location supporting brackish taxa. On the other hand, the lowest FRic values at the Krka spring may indicate that some of the resources (alpha niches) potentially available to the community are not being utilized [31]. This also means that taxa which could take advantage of these conditions are not present, removing the buffer against environmental conditions and making the system more susceptible to invasion and disturbance, which is especially important since karst springs are considered sensitive, if not endangered [81].

Caution should always be exercised in interpreting the abundance of taxa recorded by amplicon sequences, as translating abundance from sequence data to biological abundance remains a problem. Variation in rDNA copy number among taxa could be one of the main reasons for incongruent results for Alveolata sequences (ciliates), as they make up the largest proportion of sequence data [31,82]. Therefore, the number of OTUs does not reflect the same number of different taxa recorded, as the accuracy of taxonomic assignment of short amplicon sequences at the species level is still problematic due to the incompleteness of the reference database. However, our results were confirmed and compared with the microscopic analysis in the previous study [5].

## 5. Conclusions

By showing that the phylogenetic and functional diversity of ciliates change along the Krka river course, our study provides a basis for predicting the phylogenetic and functional structure of ciliates in other karstic rivers. It is not clear whether phylogenetic overdispersion at the Krka spring resulted from environmental filtering, competitive exclusion, or a combination of these factors. The higher proportion of better competitors at Krka spring compared to the downstream-most location (Skradinski buk) may have resulted in the exclusion of less competitive taxa at the former location due to the specific environmental conditions. Therefore, it is necessary to implement other methods to address this question. Given that current knowledge of functional diversity is insufficient to determine the functional niche of most protists, linking phylogenetic diversity to community patterns under the assumption of phylogenetic niche conservatism is the most precise approach we can currently use to find evidence for large-scale and community-wide processes involved in the protist communities. As the first study of its kind in the Mediterranean, our results have undoubtedly shed light on the phylogenetic and functional diversity of ciliates in karst ecosystems and provided a foundation for future ecological and conservation efforts.

## Data Availability

The data presented in this study are openly available in Sequence Read Archive of ENA at https://www.ebi.ac.uk/ena/browser/home, reference number PRJEB39359.

## Supplementary Materials

The following supporting information can be downloaded at: https://www.mdpi.com/article/10.3390/microorganisms10122493/s1, Supplementary Table S1. Functional traits of ciliates sampled at four locations (Krka spring, Marasovine, Roški slap, Skradinski buk) along the course of the Krka river, Croatia. Supplementary Table S2. Results of Tukey’s HSD parametric test applied to test location and side effect (for each location pair and side pair) for phylogenetic alpha diversity indices (PD and PSV) and functional richness metric (FRic), separately. Supplementary Table S3. Environmental parameters at the four investigated locations (Krka spring, Marasovine, Roški slap, Skradinski buk) along the course of the Krka river, Croatia. Sampling locations Krka spring (I and II), Roški slap (I and II) and Skradinski buk (I and II) were sampled on two representative habitats. Supplementary Table S4. Results of non-metric multidimensional distance scaling (NMDS) analysis of the Unifrac distance indices of the ciliate community at OTU level with environmental parameters (DIC = dissolved inorganic carbon, DOC = dissolved organic carbon, EC = conductivity, N-NO_3_^−^ = nitrates, O_2_ = oxygen saturation, pH, P-PO_4_^3−^ = phosphates, T = temperature, TN = total nitrogen). Statistically significant results (*p* < 0.05) are reported in bold.
