# Supplementary material for: Changes in Phylogenetic and Functional Diversity of Ciliates along the Course of a Mediterranean Karstic River
Source: Microorganisms. 2022 Dec 16;10(12):2493. doi: 10.3390/microorganisms10122493 (PMC9783291; doi:10.3390/microorganisms10122493)
Supplement: Supplementary file 1 [file microorganisms-10-02493-s001.zip › Supplementary Table S1.pdf]

Supplementary Table S1. Functional traits of ciliates sampled at four locations (Krka spring, Marasovine, Roški slap, Skradinski buk) along the course of the Krka river, Croatia.

Genus	Food	Feeding strategy	Habitat preference	Motility	Mode of locomotion	Life form
Acineta	omnivorous	predation	lentic and lotic (freshwater)	sessile	unknown	solitary
Aegyriana	diatoms	predation	marine	motile	gliding	solitary
Amphisiella	omnivorous	predation	soil	motile	gliding	solitary
Agolohymena	histophagous	predation	lentic and lotic (freshwater)	motile	free-swimming	solitary
Ancistrum	bacteria	unknown	symbiotic	motile	jumping, rotating	solitary
Anigsteinia	bacteria	filtration	marine	motile	gliding	solitary
Anteholosticha	omnivorous	filtration	marine	motile	crawling/creeping	solitary
Apobryophyllum	phagotrophic protists, small metazoans	predation	soil	motile	gliding	solitary
Apodileptus	omnivorous	predation	soil	motile	gliding	solitary
Apogastrostyla	diatoms	filtration	marine	motile	crawling/creeping	solitary
Aristerostoma	bacteria	unknown	marine	motile	jumping, rotating	solitary
Askenasia	algae, diatoms	filtration	lentic and lotic (freshwater)	motile	jumping, rotating	solitary

Aspidisca	bacteria	filtration	active sludge	motile	crawling/creeping	solitary
Blepharisma	algae, bacteria	filtration	lentic and lotic (freshwater)	motile	free-swimming	solitary
Bresslaua	omnivorous	predation	soil	motile	free-swimming	solitary
Bryometopus	bacteria	filtration, facultative predation	soil	motile	free-swimming	solitary
Carchesium	bacteria	filtration	lentic and lotic (freshwater)	semi-sessile	unknown	colonial
Cardiostomatella	bacteria	filtration	marine	motile	free-swimming	solitary
Chaenea	bacteria	filtration	active sludge	motile	crawling/creeping	solitary
Chilodonella	bacteria	filtration	active sludge	motile	gliding	solitary
Chlamydodon	omnivorous	unknown	marine	motile	gliding	solitary
Chlamydonella	omnivorous	predation	soil	motile	gliding	solitary
Clevelandella	omnivorous	unknown	symbiotic	motile	free-swimming	solitary
Climacostomum	omnivorous	filtration, facultative predation	lentic and lotic (freshwater)	motile	crawling/creeping	solitary
Cohnilembus	omnivorous	unknown	symbiotic	motile	free-swimming	solitary
Coleps	omnivorous	predation	lentic and lotic (freshwater)	motile	free-swimming	solitary

Colpidium	omnivorous	predation	lentic and lotic (freshwater)	motile	free-swimming	solitary
Colpoda	omnivorous	predation	soil	motile	gliding	solitary
Colpodidium	bacteria	filtration	soil	motile	jumping, rotating	solitary
Conchophthirus	omnivorous	unknown	symbiotic	motile	crawling/creeping	solitary
Condylostoma	omnivorous	filtration	soil	motile	gliding	solitary
Cryptocaryon	omnivorous	unknown	symbiotic	motile	crawling/creeping	unknown
Cultellothrix	phagotrophic protists, small metazoans	predation	soil	motile	gliding	solitary
Cyclotrichium	omnivorous	unknown	lentic and lotic (freshwater)	motile	free-swimming	solitary
Cyrtohymena	omnivorous	predation	soil	unknown	unknown	solitary
Dexiotricha	bacteria	filtration	active sludge	motile	unknown	solitary
Diaxonella	omnivorous	unknown	marine	motile	unknown	solitary
Dileptus	omnivorous	predation	soil	motile	crawling/creeping	solitary
Diophrys	omnivorous	unknown	marine	motile	crawling/creeping	solitary
Enchelys	omnivorous	predation	soil	motile	free-swimming	solitary
Engelmanniella	bacteria	unknown	soil	unknown	free-swimming	solitary
Entorhipidium	omnivorous	unknown	symbiotic	motile	crawling/creeping	solitary

Epalxella	bacteria	filtration	anaerobic mud	motile	gliding	solitary
Ephelota	omnivorous	predation	symbiotic	sessile	unknown	solitary
Epiphyllum	phagotrophic protists, small metazoans	predation	brackish	motile	gliding	solitary
Etoschophrya	omnivorous	predation	soil	motile	jumping, rotating	solitary
Euplotes	omnivorous	filtration	active sludge	motile	crawling/creeping	solitary
Euplotidium	unknown	unknown	marine	motile	unknown	solitary
Exocolpoda	bacteria	unknown	soil	motile	unknown	solitary
Fabrea	algae, bacteria	filtration	marine	motile	free-swimming	solitary
Furgasonia	algae, cyanobacteria	predation	soil	unknown	unknown	solitary
Gastrostyla	omnivorous	filtration	active sludge, soil	motile	gliding	solitary
Gonostomum	omnivorous	filtration, facultative predation	soil	motile	free-swimming	solitary
Gymnodinioides	parasitic	unknown	symbiotic	sessile	free-swimming	solitary
Halteria	omnivorous	filtration	lentic and lotic (freshwater)	motile	jumping, rotating	solitary

Hartmannula	omnivorous	unknown	marine	motile	crawling/creeping	solitary
Hemicycliostyla	omnivorous	unknown	lentic and lotic (freshwater)	motile	unknown	solitary
Hemiophrys	unknown	predation	marine	motile	gliding	solitary
Hemiurosoma	bacteria	predation	soil	motile	gliding	solitary
Heterokeronopsis	unknown	unknown	marine	motile	crawling/creeping	solitary
Histiobalantium	omnivorous	unknown	lentic and lotic (freshwater)	euplanktonic	drifting	solitary
Holosticha	omnivorous	filtration	soil	motile	free-swimming	solitary
Isotricha	bacteria	filtration	symbiotic	motile	free-swimming	solitary
Kahliella	omnivorous	unknown	soil	motile	gliding	solitary
Kentrophoros	bacteria	filtration	marine	motile	gliding	unknown
Kuklikophrya	cyanobacteria	unknown	soil	motile	gliding	solitary
Lacrymaria	phagotrophic protists, small metazoans	predation	lentic and lotic (freshwater)	motile	free-swimming	solitary
Latteuria	bacteria	unknown	symbiotic	unknown	free-swimming	unknown
Lembadion	omnivorous	filtration, facultative predation	lentic and lotic (freshwater)	motile	free-swimming	solitary
Limnostrombidium	algae	unknown	lentic and lotic (freshwater)	motile	free-swimming	solitary

Litonotus	phagotrophic protists, small metazoans	predation	soil	motile	free-swimming	solitary
Loxodes	omnivorous	predation	lentic and lotic (freshwater)	motile	free-swimming	solitary
Loxophyllum	phagotrophic protists, small metazoans	predation	lentic and lotic (freshwater)	motile	free-swimming	solitary
Maryna	bacteria	unknown	lentic and lotic (freshwater)	motile	free-swimming	solitary
Mesanophrys	parasitic	unknown	symbiotic	motile	gliding	solitary
Meseres	omnivorous	unknown	soil	motile	jumping, rotating	solitary
Mesodinium	omnivorous	unknown	lentic and lotic (freshwater)	motile	jumping, rotating	solitary
Metaurostylopsis	algae, diatoms	unknown	marine	motile	crawling/creeping	solitary
Miamiensis	histophagous	unknown	symbiotic	motile	unknown	solitary
Microxysma	algae, bacteria	unknown	marine	unknown	unknown	solitary
Nassula	algae, cyanobacteria	predation	soil	motile	free-swimming	solitary
Novistrombidium	algae	unknown	marine	motile	free-swimming	solitary
Njinella	astomata	unknown	symbiotic	unknown	unknown	unknown

Obertrumia	algae, cyanobacteria	predation	soil	motile	free-swimming	solitary
Ophryoglena	histophagous	predation	lentic and lotic (freshwater)	motile	free-swimming	solitary
Oxytricha	omnivorous	filtration	lentic and lotic (freshwater)	motile	free-swimming	solitary
Paracineta	omnivorous	predation	soil	sessile	unknown	solitary
Parafurgasonia	bacteria	predation	soil	motile	crawling/creeping	solitary
Paramecium	omnivorous	filtration	lentic and lotic (freshwater)	motile	free-swimming	solitary
Paranassula	omnivorous	predation	marine	motile	free-swimming	unknown
Paraspathidium	unknown	predation	marine	motile	crawling/creeping	solitary
Paratetrahymena	bacteria	filtration	marine	motile	gliding	solitary
Paraurostyla	omnivorous	filtration	soil	motile	free-swimming	solitary
Paruroleptus	omnivorous	unknown	soil	motile	free-swimming	solitary
Perisincirra	omnivorous	unknown	soil	motile	gliding	solitary
Phacodinium	omnivorous	unknown	soil	motile	free-swimming	solitary
Phialina	phagotrophic protists, small metazoans	predation	active sludge	motile	free-swimming	solitary
Philasterides	histophagous	unknown	lentic and lotic (freshwater)	motile	free-swimming	solitary

Placus	omnivorous	predation	lentic and lotic (freshwater)	motile	free-swimming	solitary
Plagiocampa	omnivorous	predation	soil	motile	free-swimming	solitary
Plagiopyliella	unknown	unknown	symbiotic	unknown	unknown	solitary
Platyophrya	omnivorous	unknown	soil	motile	free-swimming	solitary
Pleuronema	omnivorous	filtration	lentic and lotic (freshwater)	motile	jumping, rotating	solitary
Polydiniella	bacteria	filtration	symbiotic	unknown	unknown	unknown
Porpostoma	diatoms	filtration	symbiotic	motile	free-swimming	solitary
Prorodon	omnivorous	predation	lentic and lotic (freshwater)	motile	free-swimming	solitary
Protospathidium	phagotrophic protists, small metazoans	predation	soil	motile	free-swimming	solitary
Pseudepistylis	unknown	unknown	lentic and lotic (freshwater)	semi-sessile	gliding	colonial
Pseudochilodonopsis	algae, diatoms	predation	lentic and lotic (freshwater)	motile	unknown	solitary
Pseudocyrtolophosis	bacteria	unknown	soil	motile	crawling/creeping	solitary
Pseudokeronopsis	omnivorous	unknown	marine	motile	free-swimming	solitary
Pseudomicrothorax	algae, cyanobacteria	predation	soil	motile	free-swimming	solitary
Pseudoplatyophrya	fungi	predation	soil	motile	jumping, rotating	solitary

Pseudotontonia	unknown	unknown	marine	motile	drifting	solitary
Pseudouroleptus	omnivorous	unknown	soil	motile	gliding	solitary
Pseudourostyla	omnivorous	predation	soil	motile	gliding	solitary
Pseudovorticella	algae, bacteria	filtration	soil	semi-sessile	unknown	colonial
Raabena	unknown	unknown	symbiotic	unknown	unknown	unknown
Remanella	unknown	unknown	marine	motile	gliding	solitary
Sandmanniella	omnivorous	unknown	soil	motile	free-swimming	solitary
Schizocaryum	omnivorous	unknown	symbiotic	motile	free-swimming	unknown
Spathidiopsis	phagotrophic protists, small metazoans	predation	symbiotic	motile	free-swimming	solitary
Spirostomum	algae, bacteria	filtration	lentic and lotic (freshwater)	motile	free-swimming	solitary
Stentor	omnivorous	filtration, facultative predation	lentic and lotic (freshwater)	motile	free-swimming	solitary
Sterkiella	omnivorous	predation	active sludge, soil	motile	free-swimming	solitary
Stokesia	omnivorous	predation	lentic and lotic (freshwater)	euplanktonic	drifting	solitary

Strombidinopsis	omnivorous	unknown	marine	euplanktonic	drifting	solitary
Strombidium	omnivorous	filtration	lentic and lotic (freshwater)	motile	jumping, rotating	solitary
Strongylidium	omnivorous	unknown	soil	motile	crawling/creeping	unknown
Stylonychia	omnivorous	filtration	lentic and lotic (freshwater)	motile	free-swimming	solitary
Tetrahymena	bacteria	filtration	active sludge	motile	free-swimming	solitary
Tiarina	algae	predation	marine	motile	free-swimming	solitary
Tintinnidium	omnivorous	filtration	lentic and lotic (freshwater)	euplanktonic	drifting	solitary
Tokophrya	omnivorous	predation	soil	sessile	unknown	solitary
Trachelius	omnivorous	predation	lentic and lotic (freshwater)	motile	unknown	solitary
Trachelocerca	omnivorous	unknown	marine	motile	gliding	solitary
Trichodina	omnivorous	filtration	symbiotic	motile	jumping, rotating	unknown
Trichodinella	omnivorous	unknown	symbiotic	motile	unknown	unknown
Trichopodiella	algae, bacteria	unknown	symbiotic	motile	free-swimming	solitary
Triplumaria	unknown	unknown	symbiotic	unknown	unknown	unknown
Trithigmostoma	algae, diatoms	predation	active sludge, soil	motile	crawling/creeping	solitary
Trochilia	omnivorous	predation	active sludge	motile	crawling/creeping	solitary

Trochochilodon	unknown	unknown	marine	motile	gliding	solitary
Tunicothrix	omnivorous	unknown	marine	motile	free-swimming	solitary
Urceolaria	omnivorous	filtration	symbiotic	motile	free-swimming	solitary
Urocentrum	omnivorous	filtration	lentic and lotic (freshwater)	motile	jumping, rotating	solitary
Uroleptus	omnivorous	filtration	lentic and lotic (freshwater)	motile	free-swimming	solitary
Uronema	omnivorous	filtration	lentic and lotic (freshwater)	motile	jumping, rotating	solitary
Uronemella	bacteria	unknown	marine	motile	jumping, rotating	unknown
Urostyla	omnivorous	predation	lentic and lotic (freshwater)	motile	free-swimming	solitary
Vampyrophrya	histophagous	unknown	lentic and lotic (freshwater)	motile	free-swimming	unknown
Vorticella	omnivorous	filtration	active sludge	semi-sessile	unknown	solitary
Vorticellides	bacteria	filtration	lentic and lotic (freshwater)	semi-sessile	unknown	colonial
Woodruffides	phagotrophic protists, small metazoans	predation	soil	motile	gliding	solitary
Zosterodasys	algae, diatoms	predation	lentic and lotic (freshwater)	motile	crawling/creeping	solitary
