# Supplementary material for: Changes in Phylogenetic and Functional Diversity of Ciliates along the Course of a Mediterranean Karstic River
Source: Microorganisms. 2022 Dec 16;10(12):2493. doi: 10.3390/microorganisms10122493 (PMC9783291; doi:10.3390/microorganisms10122493)
Supplement: Supplementary file 1 [file microorganisms-10-02493-s001.zip › Supplementary Table S2.pdf]

Supplementary Table S2. Results of Tukey's HSD parametric test applied to test location and side effect (for each location pair and side pair) for phylogenetic alpha diversity indices (PD and PSV) and functional richness metric (FRic), separately.

**Metric**	**Side**	**Location pairs**	**diff**	**lwr**	**upr**	***p*val**
**PD**	light	Marasovine-Krka spring	3.370	-19.201	25.941	0.970
**PD**	light	Roski slap-Krka spring	21.200	1.873	40.531	0.029
**PD**	light	Skradinski buk-Krka spring	39.500	21.142	58.004	0.000
**PD**	light	Roski slap-Marasovine	17.800	-5.480	41.143	0.168
**PD**	light	Skradinski buk-Marasovine	36.200	13.630	58.772	0.002
**PD**	light	Skradinski buk-Roski slap	18.300	-0.959	37.698	0.066
**PD**	dark	Marasovine-Krka spring	7.460	-20.370	35.292	0.866
**PD**	dark	Roski slap-Krka spring	29.600	9.036	50.315	0.004
**PD**	dark	Skradinski buk-Krka spring	44.700	25.070	64.429	0.000
**PD**	dark	Roski slap-Marasovine	22.200	-6.303	50.732	0.155
**PD**	dark	Skradinski buk-Marasovine	37.200	9.458	65.119	0.008
**PD**	dark	Skradinski buk-Roski slap	15.070	-5.565	35.714	0.196
**PSV**	light	Roski slap-Skradinski buk	0.025	0.011	0.040	0.001
**PSV**	light	Krka spring-Skradinski buk	0.037	0.023	0.051	0.000
**PSV**	light	Marasovine-Skradinski buk	0.037	0.021	0.054	0.000
**PSV**	light	Krka spring-Roski slap	0.011	-0.003	0.026	0.149

**PSV**	light	Marasovine-Roski slap	0.012	-0.005	0.030	0.233
**PSV**	light	Marasovine-Krka spring	0.001	-0.016	0.018	0.990
**PSV**	dark	Roski slap-Skradinski buk	0.015	-0.009	0.039	0.294
**PSV**	dark	Marasovine-Skradinski buk	0.025	-0.007	0.058	0.151
**PSV**	dark	Krka spring-Skradinski buk	0.047	0.024	0.069	0.000
**PSV**	dark	Marasovine-Roski slap	0.010	-0.023	0.043	0.816
**PSV**	dark	Krka spring-Roski slap	0.031	0.008	0.055	0.009
**PSV**	dark	Krka spring-Marasovine	0.021	-0.011	0.054	0.263
**Fric**	light	Marasovine-Krka spring	2.833	-18.149	23.815	0.979
**Fric**	light	Roski slap-Krka spring	31.633	13.665	49.601	0.001
**Fric**	light	Skradinski buk-Krka spring	45.333	28.201	62.465	0.000
**Fric**	light	Roski slap-Marasovine	28.800	7.130	50.470	0.008
**Fric**	light	Skradinski buk-Marasovine	42.500	21.517	63.482	0.000
**Fric**	light	Skradinski buk-Roski slap	13.700	-4.268	31.668	0.171
**Fric**	dark	Marasovine-Krka spring	15.000	-16.679	46.667	0.538
**Fric**	dark	Roski slap-Krka spring	41.200	17.706	64.699	0.001
**Fric**	dark	Skradinski buk-Krka spring	51.500	29.099	73.901	0.000
**Fric**	dark	Roski slap-Marasovine	26.200	-6.261	58.662	0.136
**Fric**	dark	Skradinski buk-Marasovine	35.500	4.820	68.179	0.022

**Fric**	dark	Skradinski buk-Roski slap	10.300	-13.193	33.794	0.598
