# Supplementary material for: Changes in Phylogenetic and Functional Diversity of Ciliates along the Course of a Mediterranean Karstic River
Source: Microorganisms. 2022 Dec 16;10(12):2493. doi: 10.3390/microorganisms10122493 (PMC9783291; doi:10.3390/microorganisms10122493)
Supplement: Supplementary file 1 [file microorganisms-10-02493-s001.zip › Supplementary Table S3.pdf]

Supplementary Table 3. Environmental parameters at the four investigated locations (Krka spring, Marasovine, Roški slap, Skradinski buk) along the course of the Krka River, Croatia. Sampling locations Krka spring (I and II), Roški slap (I and II) and Skradinski buk (I and II) were sampled on two representative habitats.

	Krka spring I	Krka spring II	Marasovine	Roški slap I	Roški slap II	Skradinski buk I	Skradinski buk II
T (°C)	10.3	10.4	-	15.4	15.4	20.6	20.2
DO (mg L^{-1})	10.26	10.4	-	9.75	9.5	9.16	8.19
O_2 (%)	94.5	95.4	-	97.2	95.2	101.5	98.1
pH	7.75	7.76	7.88	8.35	7.96	8.58	8.53
EC (µS cm^{-1})	391	405	690	648	653	505	523
N-NO_3^- (mg L^{-1})	<0.1	<0.1	<0.1	6.6	<0.1	6.2	1.8
N-NO2^- (mg L^{-1})	<0.001	<0.001	<0.001	<0.001	<0.001	<0.001	<0.001
N-NH_4^+ (mg L^{-1})	<0.01	<0.01	<0.01	0.02	<0.01	<0.01	<0.01
P-PO_4^{3-} (mg L^{-1})	0.31	0.31	<0.01	0.27	<0.01	<0.01	<0.01
SiO_2 (mg L^{-1})	0.9	0.8	1.7	2	2.4	0.8	1.2
TN (mg L^{-1})	<0.1	<0.1	<0.1	7.1	<0.1	6.4	2
TIC (mg L^{-1})	10.77	10.78	10.46	10.79	11.06	10.55	9.78
DIC (mg L^{-1})	10.53	10.64	10.2	10.45	10.73	10.15	8.88
TOC (mg L^{-1})	0.61	1.44	0.96	0.61	0.72	1.37	2.17
DOC (mg L^{-1})	0.26	0.23	0.46	0.45	0.44	1.09	1.1
