# Supplementary material for: Changes in Phylogenetic and Functional Diversity of Ciliates along the Course of a Mediterranean Karstic River
Source: Microorganisms. 2022 Dec 16;10(12):2493. doi: 10.3390/microorganisms10122493 (PMC9783291; doi:10.3390/microorganisms10122493)
Supplement: Supplementary file 1 [file microorganisms-10-02493-s001.zip › Supplementary Table S4.pdf]

Supplementary Table 4. Results of Non-metric multidimensional distance scaling (NMDS) analysis of the Unifrac distance indices of the ciliate community at OTU level with environmental parameters (DIC = dissolved inorganic carbon, DOC = dissolved organic carbon, EC = conductivity, $N\text{-}NO_3^-$ = nitrates, O_2 = oxygen saturation, pH, $P\text{-}PO_4^{3-}$ = phosphates, T = temperature, TN = total nitrogen). Statistically significant results ($p<0.05$) are reported in bold.

	NMDS1	NMDS2	R^2	*p*
pH	0.280	-0.960	0.363	**0.002**
EC	0.674	0.738	0.120	0.098
O_2	0.135	-0.991	0.204	**0.013**
T	0.224	-0.975	0.408	**0.001**
$N\text{-}NO_3^-$	0.125	-0.992	0.320	**0.003**
$P\text{-}PO_4^{3-}$	-0.590	0.808	0.098	0.146
TN	0.135	-0.991	0.317	**0.003**
DOC	0.263	-0.965	0.249	**0.006**
DIC	-0.999	-0.033	0.140	0.062
